# Determinants of Health-Related Quality of Life in Chronic Chikungunya Disease in Guadeloupe

**DOI:** 10.3390/pathogens11090989

**Published:** 2022-08-30

**Authors:** Fabrice Simon, Rémi Bossy, Denise Federico, Julien Dezaunay, Anne-Laurence Demoux, Nadia Rugard, Giulia Calusi, Andrea Nizzardo, Hugh Watson, Franciane Gane-Troplent

**Affiliations:** 1 Emerging Virus Unit, University of Aix-Marseille, 13284 Marseille, France; 2 Faculty of Medicine Hyacinthe Bastaraud, Université des Antilles, 97157 Guadeloupe, France; 3 Pharmacometrics Department, Aptuit Srl., 37135 Verona, Italy; 4 Department of Internal Medicine, Hopital Nord, 90400 Marseille, France; 5 Antiviral Research Unit, Evotec ID, 69007 Lyon, France

**Keywords:** chikungunya virus, cohort, musculoskeletal, fatigue, sleep, depression

## Abstract

Chronic chikungunya disease is associated with a poor quality of life and a variety of symptoms, not restricted to the musculoskeletal system. Patients with chronic chikungunya disease in Guadeloupe were evaluated in order to identify the main factors determining the quality of life. Patients were followed up at a mean of 36 months after chikungunya infection, undergoing detailed clinical examination for musculoskeletal involvement, with assessment of subjective symptoms and the impact on mood, physical activity, and quality of life (SF12). Patients had extensive musculoskeletal involvement shown by tenderness in 9 ± 4 joints and stiffness in 5 ± 4 joints. SF12 physical and mental component scores showed a poor health-related quality of life. Measures of joint pain, stiffness, and inflammation contributed to impaired quality of life scores. In addition, fatigue and interrupted sleep appeared to be important predictors for physical aspects of quality of life. The emergence of anxiodepressive syndromes post-chikungunya infection was associated with both physical and mental component scores of SF12. These data confirm that musculoskeletal symptoms are not the only determinants of quality of life in chronic chikungunya disease. Follow-up of patients should include assessment and management of fatigue, poor sleep quality, and anxiodepressive syndromes.

## 1. Introduction

First identified in Tanzania in 1952, chikungunya virus (CHIKV) is an alphavirus transmitted to humans by infected *Aedes* mosquitoes. In 2004, a major outbreak in Kenya spread to islands and territories around the Indian Ocean [1]. Since then, CHIKV has been identified in up to 100 countries, with outbreaks also occurring widely in the Americas and occasionally in southern Europe [2].

CHIKV infection is responsible for a biphasic disease that generally begins 2–6 days postinfection. The acute phase of CHIKV disease is characterized by a rapid onset of fever, typically accompanied by debilitating joint pain. Acute infection often leads to chronic musculoskeletal symptoms, which may resolve only gradually over a period of months or years [3]. Persistent or recurrent arthralgia is considered the hallmark symptom of chronic chikungunya disease, but a range of different chronic manifestations have been ascribed to CHIKV infection, and the overall impact on the quality of life is considerable [4]. 

In common with some other acute viral infections [5,6], CHIKV infection is also reported to be followed by chronic fatigue and depression. Despite several studies reporting poor quality of life in patients with post-CHIKV rheumatism [7,8], the extent to which this results from musculoskeletal sequelae and how much it is due to other factors have not been determined. We investigated the factors associated with the patient’s quality of life through a comprehensive set of clinical data describing a cohort of patients with chronic chikungunya symptoms after an outbreak of CHIKV infection in the French Caribbean island of Guadeloupe. Joint pain, stiffness, and inflammation were confirmed to be associated with the quality of life scores, but other nonmusculoskeletal symptoms were also identified as contributing to impaired quality of life in these patients.

## 2. Results

One hundred and seventeen outpatients with suspected chronic chikungunya symptoms were screened. Sixty-one patients in whom CHIKV infection was confirmed serologically were enrolled into the study cohort. These patients were drawn from most parts of the island, though a majority (52.5%) came from the urban area around Pointe-á-Pitre, Les Abymes, and Le Gosier. The final study cohort of 61 patients consisted of 51 females and 10 males, with a mean age of 62 years (SD ± 11).

Patients suffered acute CHIKV infection between April 2013 and October 2015, as confirmed by detection of anti-CHIKV IgM or IgG antibodies. A clinical follow-up reported here was performed on average 36 months after the acute infection. At this time, 33/57 patients reported disturbed sleep at nights and 23/39 patients reported that they had to stop work for a period of at least 1 week during the chronic phase of the disease. Eighty-six percent of the patients also reported that they stopped all physical activity since CHIKV infection. Anxiodepressive syndromes were identified in 39 among the 57 patients responding and were considered secondary to CHIKV infection in 25 patients, but pre-existing in the other 14 cases.

The mean scores obtained on patient symptom questionnaires showed significant levels of fatigue, joint pain, and stiffness (Table 1), with notably 58% of the patients scoring the impact of joint stiffness higher than 8/10 and two-thirds of the patients with DN4 scores indicative of neuropathic pain. This was reflected in high scores for RAPID-3 and low scores for quality of life (Table 1). Physical examination revealed that joint pain was the most frequent finding with an average of more than 6/10 musculoskeletal segments affected, while joint inflammation was detected less frequently and not at all in some patients (Table 1). Forty patients had a positive metacarpal squeeze test, and 69% had a positive metatarsal squeeze test.

**Figure 1:**
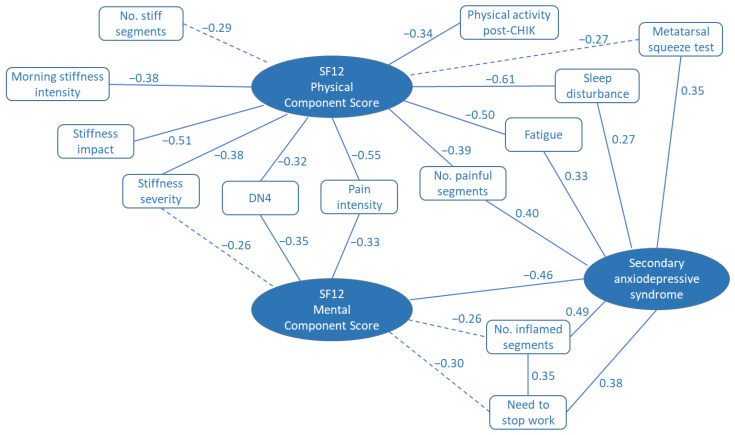
Spearman correlation map illustrating the bivariate associations between recorded variables and SF12 quality of life scores and secondary anxiodepressive syndrome. Figures provided are the Spearman rank correlation coefficients, ρ. Solid lines indicate *p* < 0.05. Broken lines indicate 0.10 > *p*> 0.05.

**Figure 2:**
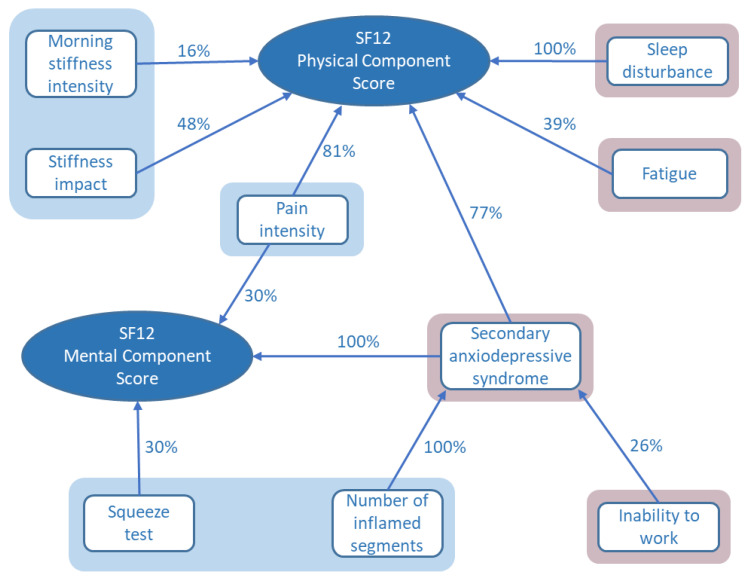
Predictors for SF12 physical and mental component scores selected by stepwise multiple linear regression. The percentages express the frequency of variable model selection on multiple imputation data sets. Measures of joint pain, stiffness, and inflammation are shown in shaded blue areas; nonmusculoskeletal factors are shaded in pink.

Fourteen variables of interest were identified from tests of bivariate associations with the SF12 physical or mental component scores (Figure 1). Thirteen of these were included in the stepwise multiple regression analysis in order to determine the most important determinants of quality of life for these patients. A fourteenth variable (physical exercise after CHIKV infection) was excluded after investigation of collinearity amongst the candidate variables. The frequency of variable selection during multiple regression analysis provides a measure of the importance of the variable in explaining the quality of life scores as depicted in Figure 2. Assessments of joint pain, stiffness, and inflammation were found to be important in the prediction of both physical and mental quality of life scores. In addition, the physical score was influenced by sleep disturbance, fatigue, and the presence of a secondary anxiodepressive syndrome. Anxiodepressive syndromes also contributed importantly to the mental component SF12 score (Figure 2).

The variables selected for the multiple regression model by this process explained 63% of the variation in SF12 physical component scores, though the statistical significance of individual variables was limited to morning stiffness (*p* = 0.05) and sleep disturbance (*p* = 0.03), with fatigue having borderline significance (*p* = 0.06). The SF12 mental component scores were less satisfactorily explained by the available data with the model accounting for only 35% of the variation in the scores. However, the role of anxiodepressive syndromes was highly significant (*p* = 0.002) with borderline significance for joint pain (*p* = 0.05).

Similar analysis performed on the variables associated with the presence of secondary anxiodepressive syndromes identified that arthritis, as measured by the number of musculoskeletal segments with inflammation, was an explanatory variable, as was the need for patients to stop their daily work for a period of at least 1 week.

## 3. Discussion

Over the last 15 years, many clinical observational studies have reported persistent musculoskeletal symptoms frequently following acute CHIKV infection, which are considered the hallmark of chronic chikungunya disease. However, there is also abundant literature reporting fatigue, sleep loss, memory and concentration difficulties, anxiety, and depression in these patients [8,9,10,11,12,13,14,15], often accompanying chronic rheumatic disorders.

Persistent symptomatic disease after CHIKV infection is associated with a reduced quality of life when measured on SF12, SF36, or EQ5D QoL scales and whether measured in the first year after the infection or some years later [7,8,9,16,17,18]. However, quantitative assessments of joint involvement in chronic chikungunya disease do not always correlate strongly with impaired quality of life [19,20] and the contribution of other chronic symptoms to the loss of quality of life has not been explored in detail.

The objective of the present study was to investigate the extent to which other features of chronic chikungunya disease may contribute to an impaired quality of life. The determinants of both physical and mental quality of life scores in our patient cohort can be divided into two groups. Consistent with the hallmark symptoms of joint pain, inflammation, and stiffness, we found that various measures of musculoskeletal symptoms contributed to explaining the quality of life scores. We also found that fatigue, disturbed sleep patterns, and the emergence of anxiodepressive syndromes may play a significant role.

Various measures of joint pain and stiffness correlated with quality of life scores. However, evidence of inflammation being associated directly with SF12 scores was restricted to the squeeze test and the mental component score of SF12. The squeeze test is a quick and easy test that may identify arthritis in the metatarsophalangeal joints [21,22]. In general, the number of involved musculoskeletal segments determined by physical examination was not the best predictor of quality of life. This is consistent with a previous study [19].

While joint inflammation is a less frequent finding in chronic chikungunya disease than painful and stiff joints, and has more limited associations with quality of life scores, the number of inflamed segments was associated with the emergence of anxiodepressive syndromes, which in turn was a predictor of both physical and mental component scores. Interrupted sleep was independently associated with physical quality of life scores in our cohort, suggesting that this phenomenon is not solely a consequence of pain and stiffness. In addition, poor sleep quality is well known to be associated with anxiodepressive syndromes, and it is tempting to see poor sleep quality, fatigue, and anxiodepressive syndromes as associated sequelae of post-chikungunya arthritis. An association of sleep disturbances with chikungunya arthritis was reported in a Colombian cohort [14]. Depression was identified during follow-up of other chikungunya outbreaks [9,12,23], and the idea of a possible mechanistic link between arthritis and depression was suggested [24] following evidence of IL6 elevation in patients with chronic rheumatic symptoms after CHIKV infection [25,26].

The association of patient symptom scores for joint pain and stiffness with quality of life scores in this cohort is consistent with the analysis of other chikungunya disease cohorts in Colombia [19] and northern Brazil [20]. Similar to those cohorts, the present study found that it was the patients’ symptom scores rather than the physician’s counts of the number of involved joints that better predicted the physical quality of life scores. In addition, the findings that other disease-related factors are also important determinants of quality of life may help us to understand why, in previous studies, impaired quality of life could only be partly explained by musculoskeletal symptoms. Interrupted sleep and need to stop work were consistently associated with physical and mental component SF12 scores, respectively, in the present cohort, and the prevalence of fatigue, which had an impact on the physical component score, has been a feature in several cohorts with chronic chikungunya disease [8,15,17,27].

A particular strength of the present study was the comprehensive assessment of patients, including physician-conducted examination, specific disease-oriented patient questionnaires, and well-characterized generic quality of life questionnaires. This permitted the investigation of a broad spectrum of factors potentially associated with the patient’s quality of life. However, certain limitations are also apparent. The cohort had a narrow age range and was predominantly female, limiting the possibility to explore any differences associated with age or sex. However, these characteristics were similar to those of many previous chronic chikungunya cohorts [9,12,18,27]. The cross-sectional design also precludes any conclusions as to the prediction of the evolution of symptoms and quality of life over time. Finally, whereas the whole cohort contributed data to univariate analyses, the final cohort size for the multiple regression analysis of quality of life was more limited due to a number of subjects with missing data for some of the variables. This limitation was mitigated by the choice of statistical model, and the conclusions are supported by findings from other chronic chikungunya cohorts. Nevertheless, the robustness of our findings should be tested prospectively in a larger cohort in the future.

Analysis of this cohort from Guadeloupe depicts the sequelae of CHIKV infection as a syndrome of musculoskeletal symptoms, fatigue, and depression associated with sleep disturbance. All of these factors were found to contribute to the poor health-related quality of life, which characterizes chronic chikungunya disease. Clinical efforts to address these aspects may result in meaningful improvements in patients’ quality of life.

## 4. Materials and Methods

### 4.1. Study Design and Setting

The setting for this study was the French Caribbean island of Guadeloupe following the chikungunya outbreak of 2013 to 2015 [28], estimated to have affected over 80,000 people in the archipelago. The target population were adults with persisting chronic symptoms after acute CHIKV infection. CHIKV infection was confirmed by IgM antibody capture enzyme-linked immunosorbent assay (ELISA) and/or indirect IgG ELISA. The study had a cross-sectional cohort design and was performed in the context of a program to improve the long-term multidisciplinary clinical management of chikungunya disease in Guadeloupe. The program, CHIK’TAMBOUYE 51, was approved by the Regional Health Agency (dossier 971-2021-06-18-00004). The study was conducted in accordance with the Declaration of Helsinki. All patients were informed about the purpose of the study and gave their consent to the inclusion of their anonymized clinical data. 

### 4.2. Clinical Data Collection

Clinical data were collected at least 36 months after the initial infection using standard questionnaires on symptoms and their impact. A musculoskeletal examination was conducted by trained physicians, who grouped joint pain, joint inflammation, joint stiffness, and muscular or tendon involvement by anatomic and functional segments. The number of segments (out of 10) affected by pain, inflammation, and stiffness was recorded, as well as the results of metacarpal and metatarsal squeeze tests to detect inflammation in the hands and feet. By means of visual analogue scales and numerical rating scales, patients recorded the degree of asthenia, joint pain, neuropathic pain on the DN4 questionnaire [29], morning stiffness, overall stiffness severity, and impact of stiffness. A global view of disease status was obtained using the RAPID-3 assessment, comprising questions on physical function, a global pain score, and a global health assessment, all answered by the patient.

The Short-Form 12 (SF12) questionnaire was used to assess the patients’ health-related quality of life, expressed as physical and mental component scores [30]. In addition, the presence of primary and secondary anxiodepressive states was recorded along with sleep disturbance and a patient’s inability to work. An anxiodepressive syndrome was defined as a patient either meeting the criteria for a major depressive episode or reporting that they suffered from mood disturbance or anxiety impacting their daily lives.

### 4.3. Statistical Methods

The characteristics of the cohort were described using means, medians, standard deviation, and interquartile ranges for the continuous variables, numbers of subjects, and percentages for categorical variables. First, the associations of the physician’s quantitative joint examination and the symptoms as perceived by the patient with the physical and mental component scores of the SF-12 quality of life questionnaire were investigated using Spearman’s correlation. Finally, multivariate analysis was performed to determine which factors were independently associated with the patients’ quality of life. This was preceded by a multicollinearity analysis including the variables selected from Spearman’s tests in order to check for any significant correlation between predictors that might justify their exclusion.

The multiple regression analysis model considered SF12 PCS, SF12 MCS, and secondary anxiodepressive syndrome as dependent variables. As predictors, 13 variables were selected as candidates from the Spearman rank correlation analyses. The most predictive independent variables were selected for each response. To reduce the impact of missing values for some variables, a multiple imputation method was used assuming that missing values are missing at random. The predictors were selected using each simulated data set by multiple linear or logistic (only for secondary anxiodepressive syndromes) regression analysis with a stepwise method on the complete data set without missing values. The higher the number of times each predictor was selected out of the total number of iterations (=1000), the more likely it was that this variable is associated with the dependent variable. To avoid biased estimates, the original data set with incomplete data was finally used to model the responses using the predictors that were selected at least 10% of the time. 

## Figures and Tables

**Table 1:** Patient symptoms, quality of life, and global disease status scores.

Parameter Assessed (Scale)	*n*	Mean ± SD	Median	IQR	Range
No. of painful segments (max 10)	61	6.2 ± 2.4	6	4	1–10
No. of stiff segments (max 10)	60	4.1 ± 2.4	3.5	4	0–10
No. of inflamed segments (max 9)	61	2.2 ± 2.0	2	3	0–9
Pain intensity (0–10)	55	6.2 ± 2.4	7	3	0–10
DN4 * (neuropathic pain) (0–10)	51	4.5 ± 2.3	4	3	0–9
Morning stiffness (0–10)	43	6.9 ± 2.6	7	4	0–10
Overall stiffness severity (0–10)	45	7.6 ± 1.7	8	3	4–10
Stiffness impact (0–10)	44	7.5 ± 2.1	8	3	1–10
Fatigue (0–100)	46	57.0 ± 24.3	60	35	0–100
SF-12 * quality of life score	45	PCS *: 32.3 ± 7.5	32.5	10.6	17.9–47.2
	45	MCS *: 34.9 ± 9.5	34.7	12.7	17.1–58.4
RAPID-3 * score (0–30)	38	17.0 ± 7.0	17.4	8.3	3 −30

* Abbreviations: DN4 = neuropathic pain scale; SF-12 = Short Form-12 item quality of lifescale; PCS = physical component score; MCS = mental component score; RAPID-3 = Routine Assessment of Patient Index Data 3.

## Data Availability

Further data supporting the reported results can be found in the thesis for Doctor of Medicine submitted by Rémi Bossy to the Faculty of Medicine Hyacinthe Bastaraud, University of the Antilles, 2020.
